# Factors Influencing Community Participation in Internet Interventions Compared With Research Trials: Observational Study in a Nationally Representative Adult Cohort

**DOI:** 10.2196/41663

**Published:** 2023-02-02

**Authors:** Philip Batterham, Amelia Gulliver, Matthew Sunderland, Louise Farrer, Frances Kay-Lambkin, Angelica Trias, Alison Calear

**Affiliations:** 1 Centre for Mental Health Research College of Health and Medicine The Australian National University Acton ACT Australia; 2 The Matilda Centre for Research in Mental Health and Substance Use University of Sydney Sydney Australia; 3 Hunter Medical Research Institute Newcastle Australia

**Keywords:** mental health, uptake, engagement, internet, research participation, implementation

## Abstract

**Background:**

Digital mental health (DMH) programs can be effective in treating and preventing mental health problems. However, community engagement with these programs can be poor. Understanding the barriers and enablers of DMH program use may assist in identifying ways to increase the uptake of these programs, which have the potential to provide broad-scale prevention and treatment in the community.

**Objective:**

In this study, we aimed to identify and compare factors that may influence participation in DMH programs in practice and research trials, identify any respondent characteristics that are associated with these factors, and assess the relationship between intentions to use DMH programs and actual uptake.

**Methods:**

Australian adults aged ≥18 years were recruited from market research panels to participate in the study. The sample was representative of the Australian adult population based on age, gender, and location. Participants completed a cross-sectional web-based survey assessing demographic characteristics, mental health symptom measures, attitudes and use of DMH programs in practice and in research studies, and the factors influencing their use in both settings.

**Results:**

Across both research and practice, trust in the organization delivering the service or trial was the top-ranked factor influencing participation, followed by anonymity or privacy and adequate information. There was little variation in rankings across demographic groups, including intentions to use DMH programs or mental health status. Intentions to use DMH programs were a strong predictor of both current (odds ratio 2.50, 99% CI 1.41-4.43; *P*<.001) and past (odds ratio 2.98, 99% CI 1.71-5.19; *P*<.001) use behaviors.

**Conclusions:**

Efforts to increase the uptake of DMH programs or participation in research trials should focus on clearly communicating the following to users: the legitimacy of the organization delivering the program, security and use of participant data, and effectiveness of DMH programs.

## Introduction

### Background

Digital mental health (DMH) programs are web-based interventions and apps that are designed to deliver evidence-based therapeutic content to individuals, with limited or no in-person clinical support. Low-intensity DMH programs, including self-help DMH programs, can be effective in treating and preventing high prevalence mental health problems such as depression or anxiety [1,2]. However, community uptake and participation in these programs remain poor [3,4], with studies in primary care reporting uptake rates as low as 3%-25% [5]. A range of factors have been proposed to explain this lack of participation, including low levels of awareness of DMH programs and the perception that web-based therapy is less effective than face-to-face care [6]. Thus, the capacity of web-based programs to provide broad-scale prevention and treatment in the community is currently limited, even though most people report that they would be willing to try a DMH program [7,8]. Identifying barriers and enablers to the use of DMH programs and developing strategies to address them are critical for optimizing their potential. An additional limitation of implementing evidence-based interventions, including both digital and in-person therapies, is that outcomes in research trials may not reflect real-world outcomes [9]. Delivery of DMH interventions in practice may not realize the success seen in controlled clinical trials [10], which typically have strict eligibility criteria and support from research staff. By examining the factors associated with engagement in research trials and comparing them with the factors associated with engagement in evidence-based interventions, we might be able to (1) better characterize implementation gaps and (2) understand differences in efficacy and use when interventions move from research to real-world delivery settings, reflecting the limited ecological validity of research trials.

### Barriers and Enablers of Engaging With Internet Interventions

Previous research has documented factors influencing the uptake (enablers) or lack of uptake (barriers) of DMH programs. Much of the previous research has described individual factors influencing *adherence* to or ongoing engagement with the program; these factors include female gender [11], sufficient time [11], less severe symptoms at baseline [12,13], younger age [12], or realization of sufficient benefit without completing the full intervention [11,12,14-16]. Research has also examined factors impeding the *uptake* or initiation of DMH programs, including a lack of familiarity or acceptability of these types of programs; data security concerns; poor attitudes toward help-seeking; assumptions that e–mental health programs are not as effective as face-to-face therapy; a lack of internet access or anxiety around its use; and other personal factors such as poor education, low knowledge of technology, personality (eg, conscientiousness), and a lack of time [2,4,5,17-22]. Much of this research, with a few recent exceptions [23,24], focuses on examining factors influencing uptake based on intervention completion data, without asking the participants themselves about their perceptions of the barriers and enablers for use. Understanding perceived barriers for potential users may provide insights into the optimal design and implementation of internet interventions. Thus, research is needed to further investigate the factors that impede the uptake of DMH programs and that are perceived to be the most important barriers preventing their use.

Some of the identified factors influencing the uptake of DMH programs may be modifiable, such as the acceptability of DMH programs [19], whereas some factors such as age or personality factors are not. We may be able to improve uptake by designing interventions that target specific groups that would benefit the most [3,13] or by challenging some of the modifiable barriers before an individual commences an e-mental health program [5]. Several studies have developed and evaluated interventions (termed *acceptance facilitation interventions or engagement facilitation interventions*) that seek to improve uptake by reducing some of these barriers [5,25,26]. These interventions have primarily focused on increasing acceptance, although some have addressed barriers such as perceived needs, privacy concerns, and knowledge [5,25,26]. Although there is evidence that these interventions can increase the acceptability of internet interventions, a recent randomized controlled trial of an engagement facilitation intervention addressing multiple barriers to engagement found no significant increase in uptake or adherence to the subsequently presented e-mental health program [27], despite aligning the design of the engagement facilitation intervention to consumer views and preferences [23]. The limited success of acceptance and engagement facilitation interventions in changing implementation outcomes suggests that engagement in interventions is highly complex and likely involves a combination of factors that interact to influence behavior. The findings also suggest that consumer preferences do not always align with their eventual behavior. However, given the limited investigation of consumer preferences and priorities to date, more work is needed to better understand the drivers of engagement with DMH programs in both research and practice, particularly in samples that are broadly representative of the general population.

### Barriers and Enablers of Engaging With Research Trials

A critical issue with DMH programs is that the findings from controlled research trials are not always consistent with outcomes when programs are implemented in clinical or community settings. Research trials benefit from the inclusion of a diverse range of participants, particularly as representation from a variety of groups increases the ability to generalize the findings to those populations [28]. However, many people may experience barriers to participation in research trials. In the general medical literature, barriers to clinical trial participation have included concerns about safety [29] or negative effects on health [30]; general fear or mistrust of medicine; the burden of trial participation [31]; and lack of access by not being offered participation [32], sometimes because of stringent inclusion criteria [33]. Enablers of participation in research trials include positive aspects of health care providers offering participation, perceived benefits, and altruism [31]. Few studies have specifically focused on factors associated with trial participation in the mental health field, although studies have examined factors influencing participation in partnership or cocreation projects, such as Living Labs [34]. From this literature, factors influencing participation in cocreation research include higher levels of socioeconomic status and more positive attitudes toward health and health care [35]. Other barriers to research engagement may include greater illness severity and functional impairments [36] and as with general health, strict eligibility criteria [37,38]. Stringent exclusion criteria are typical in mental health trials, including the trials of DMH programs, potentially resulting in highly selected samples [39]. The risk with such selection is that the effects observed in trials may not generalize when treatments are applied in real-world settings.

In summary, there is existing evidence of a range of factors influencing the uptake of DMH programs, but potential users’ perceived barriers to uptake are less frequently investigated. It also remains unclear which of these factors are the most influential in individuals’ decisions to engage or not engage with a DMH program. There has been no known previous research on the factors that influence participation in DMH research trials. In addition, previous research on factors associated with engagement has typically relied on convenience samples, which may bias the results because of underrepresentation from certain groups of the population. This study sought to fill these critical research gaps by using an Australian adult sample that was nationally representative in terms of age group, gender, and location (proportionally representing both rurality and states and territories of Australia). By comparing the similarities and differences in the factors that influence the uptake of interventions and factors associated with research participation, it may be possible to better understand the limitations of using trial data when implementing interventions in community and clinical settings.

### Aims

The primary aims of this study were to identify factors that may influence participation in DMH programs in practice and in research trials of DMH programs (aim 1) and to assess whether these factors are different (aim 2) using survey data from a representative adult sample. Putative factors were selected based on existing evidence of barriers and enablers of engagement with DMH programs and research trials. Identifying these factors in a nationally representative adult sample may inform the targeting of implementation strategies for internet interventions and may be used to identify potential limitations in the ecological validity of research trials. We also explored whether any respondent characteristics influenced how the different factors were ranked (aim 3). Finally, to examine whether the self-rated likelihood of using DMH programs was an adequate proxy for actual uptake of DMH programs, we tested the relationship between participants’ perceptions of likelihood and actual uptake (aim 4), accounting for potential confounding factors.

## Methods

### Ethics Approval

This study was approved by The Australian National University Human Research Ethics Committee (ANU HREC protocol number 2020/593). Participants were paid according to small scheduled payments for completing surveys via Qualtrics Research Services (QRS) in the form of rewards (typically used for gift cards or airline reward programs of a small value, around Aus $2 (US $1.44) to Aus $4 (US $2.88) depending on the selected reward). Data were collected anonymously, and all participants provided written informed consent before commencing the study.

Finding are reported consistently with the Checklist for Reporting Results of Internet E-Surveys (CHERRIES).

### Procedure: Recruitment and Data Collection

Participants were recruited through QRS during November 2020 to December 2020 using quota sampling to obtain a sample that was representative in terms of age group, gender, Australian state and territory populations, and remoteness (urban, regional, or rural area classification) to ensure that the sample collected was broadly representative of the Australian community. Participants from a selection of market research panels were invited by QRS to participate in the surveys. To avoid self-selection bias, survey invitations do not include specific details about the contents of the survey and are instead kept very general. After participants clicked the link from QRS, they were directed to our web page displaying the information sheet on the Qualtrics survey platform. Survey data were entered by participants on the Qualtrics platform. The inclusion criteria were that participants must be (1) living in Australia, (2) aged ≥18 years, and (3) able to read and write in English well enough to complete the survey. Participants were required to read the information sheet describing the key aspects of the study (including survey length, data storage and security, voluntary participation, the purpose of the survey, and the research team), agree that they met the inclusion criteria, and then provide consent to participate before completing the survey. Those who were screened as ineligible or did not provide their consent to participate were excluded from the study and provided with relevant mental health resources they may like to access. Participants could withdraw from the study at any time until completion by closing their web browser window, and the survey took approximately 20-30 minutes to complete. None of the questions were mandatory, except for the demographic questions used to determine the representativeness of the sample, and a *back* button was provided to review responses. Participants were excluded from the final sample if they failed to respond correctly to the 2 quality check items. QRS used IP addresses, cookies, and panel registrations to minimize repeat and poor-quality responses, excluding responses with completion time <7 minutes. If any participant endorsed the suicide screening item of the Patient Health Questionnaire-9 (PHQ-9) by selecting an option other than “not at all,” they were provided with a pop-up that asked them to telephone relevant crisis services (Lifeline, the Suicide Call Back Service, or emergency services).

### Measures

#### Overview

The usability and technical functionality of the survey were tested by the authors before it was launched. The survey questions were delivered over 15 pages, with the number of questions per page ranging from 1 to 26. Before the section on use of DMH interventions (labeled “internet-based programs”), the following definition was provided: “Internet-based programs are online learning programs or smartphone apps that provide psychological therapy strategies, coping strategies (including mindfulness or relaxation) or support for lifestyle changes to support your mental health and well-being. Internet-based programs teach strategies that are used in face-to-face therapy for reducing symptoms of depression or anxiety.” Similarly, before the section on research participation (labeled “research trials”), a definition was provided: “A research trial is a study that is run by a university, hospital or health provider, testing whether an intervention (such as a pill, device or therapy) works effectively. Participating in a research trial involves having repeated measurements (such as answering surveys or having a medical examination) and being offered a treatment, which may be an active treatment or a control (placebo) treatment. In a research trial, the treatment received by each person is determined by chance – they don't have a choice as to whether they get the active or control treatment.”

#### Demographic Characteristics

The following demographic characteristics were assessed and included in this study: gender (men, women, and nonbinary), age (18-35, 36-55, and ≥56 years), level of education (high school or less, certificate or diploma, bachelor’s degree, and postgraduate degree), language spoken at home (English and English and other, or other language only), employment status (full time, part time or casual, unemployed, not working owing to study or maternity leave, retirement, etc), and region or area of residence (metropolitan area, regional area, and rural or remote area).

#### Attitudes and Use of DMH Interventions and Research Participation

We asked participants to rate on a scale of 1 to 5 (strongly disagree=1 to strongly agree=5) the extent to which they agreed with the statements “I am confident in my ability to use the Internet,” and “I want to change the way I think and feel about mental health problems.” We also asked if they had previously used an internet-based program to support their mental health or well-being (yes and no), if they had ever participated in a research trial (yes, maybe or not sure, and no), how likely they would be to participate in a research trial in the future, and how likely they would be to try an internet intervention in 8 hypothetical situations (eg, if a clinician recommended it, out of curiosity, etc) on a 5-point scale of 1 to 5 (extremely unlikely=1 to extremely likely=5). Finally, participants were provided with links to a range of existing DMH programs (*Mindspot*, *myCompass*, and *MentalHealthOnline*), and individuals who clicked on one or more links were identified as an indicator of actual uptake.

#### Factors Influencing the Use of DMH Programs and Research Participation

We asked participants to rank a list of factors influencing community participation in web-based mental health programs in two settings: (1) use of DMH programs in practice and (2) participation in a research trial of a DMH program. These lists included items such as, “Whether I could be anonymous or maintain my privacy.” The lists were compiled based on the existing evidence of barriers and enablers of engagement in both psychosocial interventions and treatment. Before completing the lists, the participants were provided with brief definitions of internet interventions and research trials. The lists of the factors were the same, although the items were reworded to match the setting. Participants could rank all 15 factors, but we requested that participants rank at least the top 5 factors. Participants ranked at least 5 factors, with most ranking only 5 factors (use of DMH: mean 5.2, SD 1.6; research trials: mean 5.2, SD 1.5), and 13 participants providing no ranking data. To reduce the chances of response bias because of item order, we randomized the presentation order for both lists of factors and hypothetical situations mentioned previously.

#### Symptom Measures

We used the PHQ-9 [40] to measure depression, the Generalized Anxiety Disorder-7 (GAD-7) [41] to assess anxiety, and the Distress Questionnaire-5 (DQ5) [42] to measure general psychological distress. The first 2 scales assess the frequency of symptoms of major depression (PHQ-9) or generalized anxiety disorder (GAD-7) experienced during the past 2 weeks (4-point scale from not at all=0 to nearly every day=3). Item scores were summed for each scale for the overall severity scores for depression (PHQ-9:9 items; range 0-27) and anxiety (GAD-7:7 items; range 0-21) symptoms. The 5 DQ5 items asked respondents to indicate the frequency of distressing situations, thoughts, and feelings over the previous 30 days using a 5-point scale (1=never, 2=rarely, 3=sometimes, 4=often, and 5=always). Scores were summed and ranged from 5 to 25. For each of these scales, higher scores indicate higher symptom severity, and all have robust psychometric evidence for reliability and validity [42-45]. In this study, all 3 scales had high internal consistency based on Cronbach α of .93 for PHQ-9, .95 for GAD-7, and .94 for DQ5.

### Analyses

The ranked factors influencing the use of DMH programs and DMH research trial participation were reverse scored (eg, ranks 1, 2, and 3 correspond to points 3, 2, and 1, respectively) and summed so that higher scores indicated a higher ranked importance of a factor impacting participation. To compare the rankings of each factor across the 2 settings, the proportion of respondents who rated each factor as one of the top 3 factors influencing their participation was compared using McNemar test with Bonferroni correction for 15 comparisons (α=.003). Logistic regression analyses examined factors associated with high rankings (in the top 3) for the 3 most important factors associated with the 2 outcomes, with Bonferroni correction for the 6 models (α=.008). Candidate predictors of rankings included demographic factors, the likelihood of DMH program use (averaged across all scenarios), the likelihood of research trial participation, and mental health symptom scores. Finally, to examine whether the likelihood of use was related to actual uptake, we tested whether the average likelihood of DMH program use was associated with the past use of a DMH program or uptake of a DMH at the end of the survey, adjusting for demographic characteristics.

## Results

### Participant Characteristics

Table 1 presents the characteristics of the 1094 participants who completed the survey. The view rate was 31.18% (1094/3509) and the completion rate was 45% (n=3509 entered the survey, of which 117, 3.33% did not consent; 939, 26.76% did not meet demographic quotas; and 1359, 38.73% did not complete the survey or failed reliability checks). By design, the sample was representative of the Australian population in terms of age, gender, and location (both state and territory and remoteness). Although only 7.03% (77/1095) reported using the internet to support their mental health, most (655/1094, 59.87%) reported willingness to use a DMH program if it was recommended by a clinician. Most (783/1094, 71.57%) participants were somewhat or extremely likely to use a DMH program in at least one scenario. Across all scenarios, an average of 42.05% (460/1094) reported that they would be “somewhat likely” or “extremely likely” to use a DMH program. The likelihood of participating in a DMH research trial was similar, with 43.51% (476/1094) reporting that they would be “somewhat likely” or “extremely likely” to participate. Fewer than 17% (186/1094) of the participants reported prior participation in a research trial. There was a moderate correlation between the likelihood of using a DMH program and the likelihood of participating in a DMH research trial (*r*=0.31).

**Table 1:** Characteristics of participants included in the study (N=1094).

Characteristic			Participants
**Sociodemographic characteristics**			
	Age (years), mean (SD)		46.49 (18.06)
	**Age category (years), n (%)**		
		18-35	363 (33.2)
		36-55	349 (31.9)
		≥56	382 (34.9)
	**Gender, n (%)**		
		Men	529 (48.4)
		Women	561 (51.3)
		Nonbinary	4 (0.4)
	**Highest level of education, n (%)**		
		≤High school	295 (27)
		Certificate or diploma	330 (30.2)
		Bachelor’s degree	287 (26.2)
		Postgraduate degree or diploma	182 (16.6)
	**Employment, n (%)**		
		Full-time	350 (32)
		Part-time or casual	254 (23.2)
		Unemployed	108 (9.9)
		Not working (eg, study, maternity leave, etc)	382 (34.9)
	**Language, n (%)**		
		English	946 (86.5)
		English and other or other language only	148 (13.5)
	**Location, n (%)**		
		Metropolitan	789 (72.1)
		Regional	200 (18.3)
		Rural or remote	105 (9.6)
**Attitudes and use of internet interventions and research participation**			
	Previously used internet intervention for mental health, n (%)		76 (7)
	Confidence in using the internet, mean (SD)		4.33 (0.83)
	Desire to change how think and feel about mental health problems, mean (SD)		3.26 (1.09)
	**Likelihood of using internet interventions, mean (SD)**		
		If a clinician recommended that I try a program	3.46 (1.18)
		If I was diagnosed with a mental health condition	3.11 (1.24)
		If I was concerned that I had a mental health condition	3.01 (1.22)
		If a screening scale indicated I had symptoms of a mental health condition	2.98 (1.20)
		If I wanted to improve my general well-being	2.97 (1.22)
		If a friend or colleague recommended a program	2.92 (1.18)
		Out of curiosity	2.68 (1.21)
	Likelihood of participating in research trial, mean (SD)		3.17 (1.26)
	**Previously participated in research trial, n (%)**		
		Yes	108 (9.9)
		Maybe or not sure	75 (6.9)
	Uptake: clicked on one or more DMH^a^ programs, n (%)		51 (4.7)
**Mental health symptom measures, mean (SD)**			
	Anxiety (GAD-7^b^)		5.27 (5.94)
	Depression (PHQ-9^c^)		6.84 (6.95)
	General psychological distress (DQ5^d^)		10.35 (5.46)

^a^DMH: digital mental health.

^b^GAD-7: Generalized Anxiety Disorder-7.

^c^PHQ-9: Patient Health Questionnaire-9.

^d^DQ5: Distress Questionnaire-5.

### Factors Influencing Engagement With DMH Programs in Community and Research Settings

Table 2 presents the perceived factors influencing community participation in DMH programs compared with the factors influencing participation in trials of DMH programs. Trust in the organization delivering the service or trial was ranked at the top for both settings. Other factors that most highly ranked across both settings included anonymity or privacy, adequate information, and the level of need. Shame or embarrassment and other people being aware of participation or use were the lowest ranked for both settings. A direct comparison of the factors is provided in Table 3, based on the proportion of respondents who rated each factor as one of the top 3 factors influencing their participation. Two factors were more likely to be ranked highly for the use of DMH programs in practice compared with participation in DMH research trials: need for anonymity or privacy and access to other care. In contrast, 3 factors were less likely to be ranked highly in the use of DMH compared with research: need for information, effort required, and time required.

To examine whether there were characteristics associated with high rankings (top 3) of the selected factors, logistic regression models were estimated for the 3 highest-ranked items: trust, anonymity or privacy, and perceived need. The results are presented in Tables 4-6. Overall, there was little variation in rankings across demographic groups, intention to use or participate in DMH programs, and mental health status. The only significant associations were that older people were more likely to highly rank the item “Whether I trusted the organisation that delivers the program” than younger people (2% increased odds per year of age); and those who had higher intentions to use DMH programs were more likely to highly rank the item “Whether I thought I needed support for my mental health” (28% increased odds of high endorsement per one-unit increase in intentions).

**Table 2:** Factors influencing participation in digital mental health (DMH) programs and research trials.

Rank	Use of DMH programs in practice			Participation in DMH research trials	
	Factor	Score^a^	Factor		Score^a^
1	Whether I trusted the organization that delivers the program	8200	Whether I trusted the organization leading the research trial		8260
2	Whether I could be anonymous or maintain my privacy	7572	Having adequate information about the research trial		7970
3	Whether I thought I needed support for my mental health	6836	Whether I could be anonymous or maintain my privacy		6983
4	Whether the program was tailored to my needs	6643	The effort it takes to participate in the research trial		6322
5	Having information about whether the program works	5971	Whether I thought I needed support for my mental health		5954
6	Whether or not I had good access to other health services (eg, doctor, psychologist)	5217	The amount of time I have		5892
7	The effort it takes to do the program	4626	Whether the research trial was tailored to my needs		5751
8	Whether I could do the program by myself without anyone helping me	4557	Whether I had good access to health services (eg, doctor, psychologist)		4361
9	The amount of time I have	4233	My awareness of research trials		3915
10	My comfort with using technology	4030	The look, feel, and interactivity of the program being tested		3572
11	The look, feel, and interactivity of the program	3946	Whether I was assisted by a clinician to participate in the research trial		3537
12	My awareness of programs that are available	3623	My comfort with using technology		3077
13	Whether someone was going to check-in with me to complete the program	3057	Whether I could participate in the research trial by myself without anyone helping me		3075
14	Whether people I know liked the program	1945	The level of shame or embarrassment I would have about participating in a research trial		1596
15	The level of shame or embarrassment I would have about using the program	1923	Whether people I know had been in a research trial		1480

^a^Ranks reverse scored and cumulated across participants. Scores for each topic were calculated by cumulating the reverse-scored ranks for each of the 15 proposed factors (ie, ranks 1, 2, and 15 correspond to points 15, 14, and 1, respectively) across participants. Higher scores indicate higher ranked importance.

**Table 3:** Direct comparison of factors ranked in top 3.

Factor	Respondents, n (%)		McNemar *P* value
	In practice	In trials	
Trust	402 (36.7)	383 (35)	.34
Anonymity or privacy	378 (34.6)	320 (29.3)	*.002* ^a^
Need for support	327 (29.9)	283 (25.9)	.02
Tailoring	307 (28.1)	256 (23.4)	.01
Information about effectiveness	264 (24.1)	356 (32.5)	*<.001* ^a^
Access to other care	250 (22.9)	183 (16.7)	*<.001*
Effort required	193 (17.6)	292 (26.7)	*<.001*
Time available	188 (17.2)	282 (25.8)	*<.001*
Comfort with technology	184 (16.8)	148 (13.5)	.02
Ability to complete without help	180 (16.5)	139 (12.7)	.01
Awareness of availability	156 (14.3)	174 (15.9)	.26
Look and feel of program	150 (13.7)	144 (13.2)	.68
Availability of check-in or support	120 (11.0)	158 (14.4)	.01
People I know had used or participated	79 (7.2)	62 (5.7)	.12
Level of shame or embarrassment	74 (6.8)	63 (5.8)	.29

^a^Italicized values indicate *P*<.003.

**Table 4:** Logistic regression analysis of factor (trust) influencing rankings of top barriers and enablers of using digital mental health (DMH) programs in practice and participation in DMH research trials.

			Program—trust					Trial—trust		
			OR^a^ (99% CI)		*P* value		OR (99% CI)			*P* value
**Education**					.29					.25
	Certificate or diploma versus ≤HS^b^	0.864 (0.554-1.348)		.40		1.141 (0.727-1.792)			.45	
	Bachelor’s versus HS or less	0.893 (0.551-1.448)		.55		1.450 (0.896-2.346)			.047	
	Postgraduate versus HS or less	1.231 (0.714-2.121)		.33		1.276 (0.731-2.228)			.26	
**Employment**					.56					.23
	Part time or casual versus full time	0.928 (0.580-1.482)		.68		1.233 (0.772-1.970)			.25	
	Unemployed versus full time	1.054 (0.556-1.997)		.83		0.832 (0.422-1.639)			.48	
	Not in labor force versus full time	0.789 (0.484-1.286)		.21		1.304 (0.802-2.121)			.16	
Language: English only versus other			1.174 (0.703-1.961)		.42		1.218 (0.726-2.043)			.33
**Gender**					.009					.26
	Women versus men	1.400 (0.979-2.002)		.02		0.982 (0.688-1.403)			.90	
	Nonbinary versus men	11.560 (0.559-239.202)		.04		6.766 (0.327-139.971)			.10	
Age (years)			1.017 (1.004-1.031)		*.001* ^c^		1.010 (0.997-1.023)			.04
Likelihood of using DMH program			0.829 (0.678-1.013)		.02		0.972 (0.794-1.191)			.72
Likelihood of trial participation			0.956 (0.830-1.102)		.42		1.013 (0.880-1.167)			.81
PHQ-9^d^ depression score			0.998 (0.945-1.055)		.94		1.035 (0.980-1.093)			.11
GAD-7^e^ anxiety score			1.016 (0.946-1.090)		.58		0.999 (0.931-1.072)			.98
DQ5^f^ distress score			0.967 (0.897-1.041)		.24		0.974 (0.905-1.049)			.37
*Constant*			0.547 (0.145-2.072)		.24		0.226 (0.059-0.864)			.004

^a^OR: odds ratio.

^b^HS: high school.

^c^Italicized values indicate *P*<.008.

^d^PHQ-9: Patient Health Questionnaire-9.

^e^GAD-7: Generalized Anxiety Disorder-7.

^f^DQ5: Distress Questionnaire-5.

**Table 5:** Logistic regression analysis of factor (privacy) influencing rankings of top barriers and enablers of using digital mental health (DMH) programs in practice and participation in DMH research trials.

		Program—privacy		Trial—privacy	
		OR^a^ (99% CI)	*P* value	OR (99% CI)	*P* value
**Education**			.68		.16
	Certificate or diploma versus ≤HS^b^	1.011 (0.650-1.571)	.95	1.164 (0.731-1.854)	.40
	Bachelor’s versus ≤HS	1.027 (0.637-1.655)	.89	1.294 (0.786-2.131)	.18
	Postgraduate versus ≤HS	0.803 (0.454-1.421)	.32	0.808 (0.439-1.484)	.37
**Employment**			.08		.15
	Part time or casual versus full time	0.983 (0.608-1.590)	.93	0.698 (0.423-1.152)	.07
	Unemployed versus full time	1.341 (0.714-2.521)	.23	1.054 (0.550-2.018)	.84
	Not in labor force versus full time	1.519 (0.933-2.473)	.03	1.071 (0.648-1.771)	.72
Language: English only versus other		1.444 (0.846-2.465)	.08	1.065 (0.624-1.820)	.76
**Gender**			.80		.07
	Women versus Men	1.001 (0.700-1.433)	.99	1.291 (0.889-1.875)	.08
	Nonbinary versus men	0.449 (0.021-9.615)	.50	6.557 (0.317-135.544)	.11
Age (years)		0.995 (0.983-1.008)	.34	0.997 (0.983-1.010)	.51
Likelihood of using DMH program		0.919 (0.749-1.126)	.28	0.862 (0.697-1.066)	.07
Likelihood of trial participation		1.117 (0.969-1.286)	.04	1.049 (0.905-1.215)	.41
PHQ-9^c^ depression score		1.033 (0.979-1.091)	.12	1.009 (0.954-1.067)	.67
GAD-7^d^ anxiety score		0.997 (0.930-1.069)	.91	0.994 (0.925-1.069)	.84
DQ5^e^ distress score		0.992 (0.921-1.067)	.77	1.015 (0.941-1.095)	.61
*Constant*		0.337 (0.088-1.286)	.04	0.424 (0.106-1.700)	.11

^a^OR: odds ratio.

^b^HS: high school.

^c^PHQ-9: Patient Health Questionnaire-9.

^d^GAD-7: Generalized Anxiety Disorder-7.

^e^DQ5: Distress Questionnaire-5.

**Table 6:** Logistic regression analysis of factor (need) influencing rankings of top barriers and enablers of using digital mental health (DMH) programs in practice and participation in DMH research trials.

			Program—need					Trial—need		
			OR^a^ (99% CI)		*P* value		OR (99% CI)			*P* value
**Education**					.91					.16
	Certificate or diploma versus ≤HS^b^	1.032 (0.650-1.640)		.86		1.063 (0.663-1.703)			.74	
	Bachelor’s versus ≤HS	0.924 (0.559-1.527)		.69		0.696 (0.407-1.189)			.08	
	Postgraduate versus ≤HS	0.901 (0.502-1.619)		.65		0.809 (0.439-1.490)			.37	
**Employment**					.97					.81
	Part time or casual versus full time	1.078 (0.658-1.767)		.70		0.936 (0.551-1.591)			.75	
	Unemployed versus full time	0.953 (0.477-1.904)		.86		0.923 (0.448-1.904)			.78	
	Not in labor force versus full time	1.027 (0.619-1.706)		.89		1.128 (0.665-1.912)			.56	
Language: English only versus other			1.389 (0.798-2.418)		.13		1.265 (0.705-2.270)			.30
**Gender**					.87					.95
	Women versus men	1.069 (0.736-1.551)		.65		0.952 (0.645-1.405)			.74	
	Nonbinary versus men	1.405 (0.067-29.400)		.77		0.000 (indeterminate)			.99	
Age (years)			1.006 (0.993-1.020)		.23		1.006 (0.993-1.019)			.28
Likelihood of using DMH program			1.281 (1.029-1.594)		*.004* ^c^		1.187 (0.949-1.486)			.05
Likelihood of trial participation			1.110 (0.957-1.288)		.07		1.144 (0.980-1.334)			.03
PHQ-9^d^ depression score			0.980 (0.924-1.040)		.39		0.945 (0.886-1.007)			.02
GAD-7^e^ anxiety score			0.992 (0.918-1.071)		.78		1.038 (0.958-1.124)			.23
DQ5^f^ distress score			0.981 (0.907-1.061)		.52		0.990 (0.912-1.075)			.75
*Constant*			0.120 (0.029-0.495)		<.001		0.132 (0.030-0.578)			<.001

^a^OR: odds ratio.

^b^HS: high school.

^c^Italicized values indicate *P*<.008.

^d^PHQ-9: Patient Health Questionnaire-9.

^e^GAD-7: Generalized Anxiety Disorder-7.

^f^DQ5: Distress Questionnaire-5.

### Relationship Between DMH Use Intentions and Behaviors

Finally, to examine whether the self-rated likelihood of using DMH programs was a good proxy for actual uptake of DMH programs, we estimated 2 logistic regressions: one on past use of DMH programs and one on uptake of a DMH program within the study. The results of these analyses are presented in Table 7. The analyses indicated that use intentions were a strong indicator of both current and past behaviors. Specifically, there was 2.5-fold increase in the odds of engaging with one of the offered DMH programs per one-point increase in intentions and nearly 3-fold increase in the odds of past DMH program use per one-point increase in intentions. Younger age was also significantly associated with the past use of DMH programs, with a 6% increase in the odds of past use per year of age.

**Table 7:** Logistic regression analyses testing the associations between use intentions and behaviors.

			New uptake of DMH^a^ program				Previous use of DMH program		
			OR^b^ (99% CI)		*P* value		OR (99% CI)		*P* value
Likelihood of using internet intervention			2.503 (1.413, 4.434)		*<.001* ^c^		2.982 (1.714, 5.187)		*<.001* ^c^
**Education**					.34				.76
	Certificate or diploma versus ≤HS^d^	1.663 (0.615, 4.498)		.19		1.182 (0.482, 2.903)		.63	
	Bachelor’s degree versus ≤HS	0.968 (0.312, 3.002)		.94		0.841 (0.338, 2.090)		.62	
	Postgraduate degree versus ≤HS	0.850 (0.207, 3.487)		.77		1.171 (0.387, 3.546)		.71	
**Employment**					.57				.39
	Part time or casual versus full time	0.967 (0.322, 2.907)		.94		0.924 (0.380, 2.251)		.82	
	Unemployed versus full time	0.924 (0.195, 4.375)		.90		1.442 (0.485, 4.290)		.39	
	Not in labor force versus full time	1.607 (0.548, 4.717)		.26		1.648 (0.639, 4.250)		.17	
Language: English only versus other			0.680 (0.229, 2.025)		.36		1.226 (0.489, 3.074)		.57
Gender: Male versus other			2.023 (0.867, 4.721)		.03		0.779 (0.388, 1.562)		.36
Age (years)			1.013 (0.987, 1.040)		.20		0.942 (0.918, 0.967)		*<.001* ^c^
*Constant*			0.001 (1.413, 4.434)		*<.001*		0.018 (1.714, 5.187)		*<.001*

^a^DMH: digital mental health.

^b^OR: odds ratio.

^c^Italicized values indicate *P*<.001.

^d^HS: high school.

## Discussion

### Principal Findings

The findings indicate that among a representative Australian adult sample, a few key factors are the common influences of engagement with DMH programs in the context of both use in practice and in research trials. Although these factors may be considered as either “barriers” or “enablers” of uptake, each factor represents both a challenge to clearly articulate the potential difficulties in implementing DMH programs and an opportunity to educate and engage the target audience (ie, both a potential barrier and enabler). The most highly ranked considerations that influence the use of DMH programs were trust in the provider, need for privacy or anonymity, perceived need for support, and information about whether a DMH program is effective.

These outcomes differ somewhat from those of our previous community-based study in which sufficient time and perceived need dominated as key barriers to engagement [23]. Although perceived need was important in this study, the role of trust and need for privacy ranked higher, whereas time constraints had much less influence on use intentions. There are a few possible explanations for the discrepancies. First, we used a ranking approach rather than assigning equal weight to all reported barriers or enablers. Second, our focus was on the uptake or initiation of an intervention rather than on ongoing engagement. In agreement with this, a recent study on university medical students’ attitudes specifically toward the uptake of DMH found that privacy was also a key barrier to engaging in this group [24]. Third, barriers may change over time. For example, public awareness of privacy issues may have increased in recent years in response to problems with electronic medical records or media stories of hacking. Finally, our sample was selected to be representative of the national adult population in Australia in terms of age, gender and location, whereas most previous research on the factors influencing DMH use has relied on convenience samples. It is possible that convenience samples overrepresent people with mental health problem [46] or result in highly educated samples [47] that may have greater existing knowledge of DMH programs. Such biases may skew the preferences and priorities of a sample, leading to conclusions that may neglect the perspectives of underrepresented groups of the community. For example, the mean depression and anxiety symptom scores in this sample were approximately half of those seen in convenience samples [3].

The roles of trust and the need for privacy have been highlighted in previous studies [48-50] and are 2 themes that are likely to intersect [51]. Trust in the provider was the most important consideration both when conducting research trials and when delivering a DMH program in practice. This finding suggests that there may be not only challenges in building trust between providers and end users but also opportunities to promote DMH programs through trusted brands within the mental health sector. Organizations with established reputations for rigor, such as universities, may be trusted more by the public than commercial organizations [52]. Partnerships between stakeholders to ensure that DMH programs meet rigorous standards of safety and efficacy, combined with the reach and infrastructure of the industry and government, may be an important approach for further implementing DMH programs into practice. Privacy and data security have also been identified as key concerns. Clarity around what data are collected and how they are used is likely to be important in promoting uptake, along with emphasizing when programs are delivered anonymously. This information is often lacking or substandard in existing services [48].

The participants also ranked the need for sufficient information on the effectiveness of DMH programs as a key determinant of their use. Marketing DMH programs may benefit from presenting information about the effectiveness of the program in a way that is suitable for diverse audiences [53], without overstating or neglecting existing evidence [23]. Perceived need may be more challenging to address, as it is difficult for programs to engage people experiencing mental health problems if they are unaware that they may benefit from support. Strategies to address this challenge include the implementation of community mental health literacy programs [54], population-based screening with feedback [53,55], and implementation and marketing of DMH programs in diverse clinical and community settings [56]. Novel and effective approaches to research dissemination into the community may also raise awareness of the availability and evidence base of DMH programs. Encouragingly, stigma and embarrassment, along with the perceptions of others, were the least endorsed factors, suggesting that negative attitudes toward people with mental illness and negative attitudes toward treatment may have limited influence on the uptake of digital interventions.

Despite many similarities, there were a few differences in rankings between the use of DMH programs in practice and research. The need for anonymity and privacy was found to be more important for engaging with DMH programs in practice. Having access to other care was also more relevant to DMH in practice. Interestingly, the effort and time required were seen as more important in research trials, indicating that people view research as more burdensome than engaging with a service. This finding may be related to the additional burden of engaging with a research team and completing assessments in the context of a trial. These findings suggest that research cohorts may attract people with greater time and capacity than those who might otherwise engage in DMH programs. Such differences in the profiles of research participants may distort the trial outcomes when DMH programs are implemented in clinical or community settings. Furthermore, information needs were ranked higher in research trials, emphasizing the importance of clearly communicating expectations around participation in trials.

The rankings varied little by demographic characteristics, level of intention to use DMH programs, or mental health symptoms, suggesting that the perceived barriers and enablers to use are relatively homogeneous across the general population. Older adults were more likely to endorse trust as a key concern, suggesting that the reputation of a provider becomes increasingly important when delivering services to older adults. Those who reported a greater likelihood of using DMH programs were more likely to endorse perceived need as a determinant of use, which may indicate that those who are most likely to use programs tend to consider the level of support is needed (eg, no care, informal care, DMH programs, or face-to-face services) based on the severity of their symptoms. However, other demographic factors and mental health symptoms did not influence the relative importance of each factor.

Research on preferences for interventions is sometimes criticized for using behavioral intentions as a proxy for actual behavior. However, we also demonstrated that the likelihood of using a DMH program was strongly associated with both past and current engagement in these programs. These findings suggest that behavioral intentions are likely to be a good proxy for future use, as each 1-point increase in intentions was associated with 2.5-times the odds of engaging with one of the three DMH programs that were offered at the end of the survey. The findings also indicate that people who had engaged in DMH programs in the past had higher intentions for future use, which is reassuring as it suggests a level of satisfaction with past experiences.

### Limitations

This study is one of very few to use a nationally representative adult sample to examine the factors associated with engagement in DMH programs. It is also the first, to our knowledge, to concurrently examine barriers and enablers of using DMH programs in practice and in research trials, providing useful information about why trial outcomes may not translate into practice. However, some limitations of this study should be acknowledged. Although we recruited the sample to be representative of key demographic characteristics, selection biases may still exist, such as those related to access to technology, interest in research, and interest in mental health. Potential participants may have withdrawn (or may have been more inclined to participate) when provided with information about the content of the survey, and some may have been motivated to participate by the small incentive. Furthermore, market research panels may not be representative of the community in terms of other characteristics. In particular, panel members may be more accustomed to research, more familiar with digital applications, and have potentially more favorable attitudes toward research than people who do not engage in such panels. As it is extremely challenging to engage research-averse people in research studies, methods to account for nonparticipation [57] may provide further insight into barriers to service engagement. The study was cross-sectional; therefore, the direction of the effects and causation cannot be inferred. Our indicators of the likelihood of using DMH programs were ad hoc, using hypothetical scenarios that may not reflect actual use or specific programs. Nevertheless, these indicators were strongly related to self-reported past use and the choice to engage in a DMH program at the end of the survey. It is also acknowledged that clicking on the link to a DMH program signifies behavior toward using a program, rather than direct engagement with program content, and factors such as previous experience using DMH programs may have influenced the decision to click on links. Our choice of barriers and enablers associated with the decision to engage in a DMH program was intended to be comprehensive, covering the most commonly endorsed factors identified in previous research. However, there may be other factors that we did not consider. Finally, other unmeasured factors such as personality, access to resources, socioeconomic status, and ethnicity may also influence the observed associations.

### Conclusions

The factors that influence engagement in DMH programs are largely consistent in the context of both use in practice and research trials, with some key exceptions. Trust in providers, need for privacy or anonymity, perceived need for support, and information about the effectiveness of DMH programs were commonly endorsed in both settings, whereas time commitment was a greater consideration for research trials than for engaging in DMH programs in practice. Bridging the implementation gap between evidence and the use of DMH programs requires clearly communicating to users the legitimacy of the organization delivering the program. Organizations that deliver DMH programs are often successful at either developing evidence for efficacy and safety or marketing a program but not always both [58]. The findings of this study, in the context of growing literature on the implementation of DMH programs, suggest several additional considerations for optimizing the implementation of DMH programs, including providing transparent information about data use, demonstrating the effectiveness of the program, ensuring that programs are developed around the needs of end users [58], and providing clearer dissemination pathways with better marketing of evidence-based programs in community and clinical settings [56].

## Abbreviations

DMH: digital mental health
DQ5: Distress Questionnaire-5
GAD-7: Generalized Anxiety Disorder-7
PHQ-9: Patient Health Questionnaire-9
QRS: Qualtrics Research Services

## Data Availability

Data are available on request from the primary author.

## References

[1] Karyotaki E, Riper H, Twisk J, Hoogendoorn A, Kleiboer A, Mira A, Mackinnon A, Meyer B, Botella C, Littlewood E, Andersson G, Christensen H, Klein JP, Schröder J, Bretón-López J, Scheider J, Griffiths K, Farrer L, Huibers MJ, Phillips R, Gilbody S, Moritz S, Berger T, Pop V, Spek V, Cuijpers P (2017). Efficacy of self-guided internet-based cognitive behavioral therapy in the treatment of depressive symptoms: a meta-analysis of individual participant data. JAMA Psychiatry.

[2] Batterham PJ, Sunderland M, Calear AL, Davey CG, Christensen H, Teesson M, Kay-Lambkin F, Andrews G, Mitchell PB, Herrman H, Butow PN, Krouskos D (2015). Developing a roadmap for the translation of e-mental health services for depression. Aust N Z J Psychiatry.

[3] Batterham PJ, Calear AL (2017). Preferences for Internet-based mental health interventions in an adult online sample: findings from an online community survey. JMIR Ment Health.

[4] Musiat P, Goldstone P, Tarrier N (2014). Understanding the acceptability of e-mental health--attitudes and expectations towards computerised self-help treatments for mental health problems. BMC Psychiatry.

[5] Ebert DD, Berking M, Cuijpers P, Lehr D, Pörtner M, Baumeister H (2015). Increasing the acceptance of internet-based mental health interventions in primary care patients with depressive symptoms. A randomized controlled trial. J Affect Disord.

[6] Apolinário-Hagen J, Harrer M, Kählke F, Fritsche L, Salewski C, Ebert DD (2018). Public attitudes toward guided internet-based therapies: web-based survey study. JMIR Ment Health.

[7] Clough BA, Zarean M, Ruane I, Mateo NJ, Aliyeva TA, Casey LM (2019). Going global: do consumer preferences, attitudes, and barriers to using e-mental health services differ across countries?. J Ment Health.

[8] Choi I, Sharpe L, Li S, Hunt C (2015). Acceptability of psychological treatment to Chinese- and Caucasian-Australians: internet treatment reduces barriers but face-to-face care is preferred. Soc Psychiatry Psychiatr Epidemiol.

[9] Heneghan C, Goldacre B, Mahtani KR (2017). Why clinical trial outcomes fail to translate into benefits for patients. Trials.

[10] Andersson G (2018). Internet interventions: past, present and future. Internet Interv.

[11] Batterham PJ, Neil AL, Bennett K, Griffiths KM, Christensen H (2008). Predictors of adherence among community users of a cognitive behavior therapy website. Patient Prefer Adherence.

[12] Christensen H, Griffiths KM, Farrer L (2009). Adherence in internet interventions for anxiety and depression. J Med Internet Res.

[13] Farrer LM, Griffiths KM, Christensen H, Mackinnon AJ, Batterham PJ (2014). Predictors of adherence and outcome in internet-based cognitive behavior therapy delivered in a telephone counseling setting. Cogn Ther Res.

[14] Davis MJ, Addis ME (1999). Predictors of attrition from behavioral medicine treatments. Ann Behav Med.

[15] Donkin L, Christensen H, Naismith SL, Neal B, Hickie IB, Glozier N (2011). A systematic review of the impact of adherence on the effectiveness of e-therapies. J Med Internet Res.

[16] Donkin L, Glozier N (2012). Motivators and motivations to persist with online psychological interventions: a qualitative study of treatment completers. J Med Internet Res.

[17] (2015). Australian Government Response to Contributing Lives, Thriving Communities: Review of Mental Health Programmes and Services. Australian Government Department of Health.

[18] Gun SY, Titov N, Andrews G (2011). Acceptability of Internet treatment of anxiety and depression. Australas Psychiatry.

[19] Waller R, Gilbody S (2009). Barriers to the uptake of computerized cognitive behavioural therapy: a systematic review of the quantitative and qualitative evidence. Psychol Med.

[20] Mohr DC, Siddique J, Ho J, Duffecy J, Jin L, Fokuo JK (2010). Interest in behavioral and psychological treatments delivered face-to-face, by telephone, and by internet. Ann Behav Med.

[21] Casey LM, Wright MA, Clough BA (2014). Comparison of perceived barriers and treatment preferences associated with internet-based and face-to-face psychological treatment of depression. Int J Cyber Behav Psychol Learn.

[22] Gulliver A, Calear AL, Sunderland M, Kay-Lambkin F, Farrer LM, Batterham PJ (2021). Predictors of acceptability and engagement in a self-guided online program for depression and anxiety. Internet Interv.

[23] Gulliver A, Calear AL, Sunderland M, Kay-Lambkin F, Farrer LM, Banfield M, Batterham PJ (2020). Consumer-guided development of an engagement-facilitation intervention for increasing uptake and adherence for self-guided Web-based mental health programs: focus groups and online evaluation survey. JMIR Form Res.

[24] Dederichs M, Weber J, Pischke CR, Angerer P, Apolinário-Hagen J (2021). Exploring medical students' views on digital mental health interventions: a qualitative study. Internet Interv.

[25] Baumeister H, Seifferth H, Lin J, Nowoczin L, Lüking M, Ebert D (2015). Impact of an acceptance facilitating intervention on patients' acceptance of internet-based pain interventions: a randomized controlled trial. Clin J Pain.

[26] Baumeister H, Nowoczin L, Lin J, Seifferth H, Seufert J, Laubner K, Ebert DD (2014). Impact of an acceptance facilitating intervention on diabetes patients' acceptance of internet-based interventions for depression: a randomized controlled trial. Diabetes Res Clin Pract.

[27] Batterham PJ, Calear AL, Sunderland M, Kay-Lambkin F, Farrer LM, Christensen H, Gulliver A (2021). A brief intervention to increase uptake and adherence of an internet-based program for depression and anxiety (enhancing engagement with psychosocial interventions): randomized controlled trial. J Med Internet Res.

[28] Britton A, McKee M, Black N, McPherson K, Sanderson C, Bain C (1999). Threats to applicability of randomised trials: exclusions and selective participation. J Health Serv Res Policy.

[29] Lewin J, Bell JA, Wang K, Forcina V, Tam S, Srikanthan A, Lin YC, Taback N, Mitchell L, Gupta AA (2020). Evaluation of adolescents' and young adults' attitudes toward participation in cancer clinical trials. JCO Oncol Pract.

[30] Abdelhafiz AS, Abd ElHafeez S, Khalil MA, Shahrouri M, Alosaim B, Salem RO, Alorabi M, Abdelgawad F, Ahram M (2021). Factors influencing participation in COVID-19 clinical trials: a multi-national study. Front Med (Lausanne).

[31] Schmotzer GL (2012). Barriers and facilitators to participation of minorities in clinical trials. Ethn Dis.

[32] Wendler D, Kington R, Madans J, Van Wye G, Christ-Schmidt H, Pratt LA, Brawley OW, Gross CP, Emanuel E (2006). Are racial and ethnic minorities less willing to participate in health research?. PLoS Med.

[33] Comis RL, Miller JD, Aldigé CR, Krebs L, Stoval E (2003). Public attitudes toward participation in cancer clinical trials. J Clin Oncol.

[34] Georges A, Schuurman D, Baccarne B, Coorevits L (2015). User engagement in living lab field trials. Info.

[35] Hearld KR, Hearld LR, Hall AG (2017). Engaging patients as partners in research: factors associated with awareness, interest, and engagement as research partners. SAGE Open Med.

[36] Lally J, Watkins R, Nash S, Shetty H, Gardner-Sood P, Smith S, Murray RM, Gaughran F (2018). The representativeness of participants with severe mental illness in a psychosocial clinical trial. Front Psychiatry.

[37] Hoertel N, Le Strat Y, Blanco C, Lavaud P, Dubertret C (2012). Generalizability of clinical trial results for generalized anxiety disorder to community samples. Depress Anxiety.

[38] Hoertel N, Le Strat Y, De Maricourt P, Limosin F, Dubertret C (2013). Are subjects in treatment trials of panic disorder representative of patients in routine clinical practice? Results from a national sample. J Affect Disord.

[39] Lorenzo-Luaces L, Johns E, Keefe JR (2018). The generalizability of randomized controlled trials of self-guided internet-based cognitive behavioral therapy for depressive symptoms: systematic review and meta-regression analysis. J Med Internet Res.

[40] Spitzer RL, Kroenke K, Williams JB (1999). Validation and utility of a self-report version of PRIME-MD: the PHQ primary care study. Primary care evaluation of mental disorders. Patient health questionnaire. JAMA.

[41] Spitzer RL, Kroenke K, Williams JB, Löwe B (2006). A brief measure for assessing generalized anxiety disorder: the GAD-7. Arch Intern Med.

[42] Batterham PJ, Sunderland M, Carragher N, Calear AL, Mackinnon AJ, Slade T (2016). The Distress Questionnaire-5: population screener for psychological distress was more accurate than the K6/K10. J Clin Epidemiol.

[43] Kroenke K, Spitzer RL, Williams JB, Löwe B (2010). The patient health questionnaire somatic, anxiety, and depressive symptom scales: a systematic review. Gen Hosp Psychiatry.

[44] Löwe B, Decker O, Müller S, Brähler E, Schellberg D, Herzog W, Herzberg PY (2008). Validation and standardization of the Generalized Anxiety Disorder Screener (GAD-7) in the general population. Med Care.

[45] Batterham PJ, Sunderland M, Slade T, Calear AL, Carragher N (2018). Assessing distress in the community: psychometric properties and crosswalk comparison of eight measures of psychological distress. Psychol Med.

[46] Batterham PJ (2014). Recruitment of mental health survey participants using Internet advertising: content, characteristics and cost effectiveness. Int J Methods Psychiatr Res.

[47] Späth C, Hapke U, Maske U, Schröder J, Moritz S, Berger T, Meyer B, Rose M, Nolte S, Klein JP (2017). Characteristics of participants in a randomized trial of an Internet intervention for depression (EVIDENT) in comparison to a national sample (DEGS1). Internet Interv.

[48] O'Loughlin K, Neary M, Adkins EC, Schueller SM (2019). Reviewing the data security and privacy policies of mobile apps for depression. Internet Interv.

[49] Gulliver A, Bennett K, Bennett A, Farrer LM, Reynolds J, Griffiths KM (2015). Privacy issues in the development of a virtual mental health clinic for university students: a qualitative study. JMIR Ment Health.

[50] Ritterband LM, Gonder-Frederick LA, Cox DJ, Clifton AD, West RW, Borowitz SM (2003). Internet interventions: in review, in use, and into the future. Prof Psychol Res Pr.

[51] Iott BE, Campos-Castillo C, Anthony DL (2020). Trust and privacy: how patient trust in providers is related to privacy behaviors and attitudes. AMIA Annu Symp Proc.

[52] LaMonica HM, Davenport TA, Roberts AE, Hickie IB (2021). Understanding technology preferences and requirements for health information technologies designed to improve and maintain the mental health and well-being of older adults: participatory design study. JMIR Aging.

[53] Batterham PJ, Calear AL, Farrer L, Gulliver A, Kurz E (2021). Efficacy of a transdiagnostic self-help internet intervention for reducing depression, anxiety, and suicidal ideation in adults: randomized controlled trial. J Med Internet Res.

[54] Brijnath B, Protheroe J, Mahtani KR, Antoniades J (2016). Do web-based mental health literacy interventions improve the mental health literacy of adult consumers? Results from a systematic review. J Med Internet Res.

[55] Batterham PJ, Calear AL, Sunderland M, Carragher N, Brewer JL (2016). Online screening and feedback to increase help-seeking for mental health problems: population-based randomised controlled trial. BJPsych Open.

[56] Batterham PJ, Gulliver A, Kurz E, Farrer LM, Vis C, Schuurmans J, Calear AL (2022). The effect of dissemination pathways on uptake and relative costs for a transdiagnostic, self-guided internet intervention for reducing depression, anxiety, and suicidal ideation: comparative implementation study. J Med Internet Res.

[57] Bergstrand R, Vedin A, Wilhelmsson C, Wilhelmsen L (1983). Bias due to non-participation and heterogenous sub-groups in population surveys. J Chronic Dis.

[58] Torous J, Nicholas J, Larsen ME, Firth J, Christensen H (2018). Clinical review of user engagement with mental health smartphone apps: evidence, theory and improvements. Evid Based Ment Health.

